# Urine and Saliva: Relevant Specimens for Malaria Diagnosis?

**DOI:** 10.3390/diagnostics12122989

**Published:** 2022-11-29

**Authors:** Hwa Chia Chai, Kek Heng Chua

**Affiliations:** Department of Biomedical Science, Faculty of Medicine, University of Malaya, Kuala Lumpur 50603, Malaysia

**Keywords:** urine, saliva, malaria, rapid diagnostic tests, nucleic acid, biomarkers

## Abstract

Blood remains the specimen of preference for malaria diagnosis, whether it is for microscopic, nucleic acid-based or biomarker detection of *Plasmodium* present in a patient. However, concerning the disadvantages of blood drawing, specimens that can be non-invasively collected under non-hygienic settings would come in handy for malaria diagnosis in endemic areas with limited resources. Although the current approaches using saliva or urine might not be as sensitive and specific as using blood, the potential of these two specimens should not be underestimated and efforts in developing diagnostic methods for *Plasmodium* detection specifically in these two specimens should continue without giving up. This review not only compiles and summarizes the sensitivity and specificity achieved by various detection approaches when using these samples for malaria diagnosis, it also intends to enhance the possibility of using saliva and urine for diagnostic purposes by describing how *Plasmodium* nucleic acid and antigens may likely be present in these samples. This review may hopefully encourage and motivate researchers in developing saliva- and urine-based diagnostic methods for *Plasmodium* detection to facilitate the control and eradication of malaria. In summary, the presence of *Plasmodium* DNA and antigens in urine and saliva makes these two specimens relevant and useful for malaria diagnosis.

## 1. Introduction

The current malaria diagnosis, regardless of whether the diagnosis is via microscopic examination, rapid diagnostic test (RDT), or nucleic acid-based approach, still relies highly on blood collection. Blood products, including serum and plasma, are by far the most well-accepted choice of specimen since blood circulates around all tissues and organs and most likely carries with it the by-products from diseased areas as well as the pathogens themselves and their antigens [1]. However, the invasive procedure of blood collection must be performed by trained personnel and it could be an issue for some individuals, especially children, people with trypanophobia and communities having blood taboos [2]. Blood drawing may be associated with side effects such as bruising or pain and hematoma, but sometimes more serious complications such as accidental infection may occur, particularly at resource-poor field settings [3,4]. In addition, vulnerable patients may lose compliance when repeated sampling is required [5] and this could hinder the continuous surveillance of malaria diagnosis or treatment due to limited enrolment of participants in biomedical research [6]. Therefore, non-invasive and rapid malaria diagnostic tools using other body fluids are invaluable for healthcare delivery, especially in peripheral settings.

Both urine and saliva are attractive body fluids to be explored for their potential use in disease biomarker detection or diagnosis. No special equipment is needed for collection of both types of specimens and hence they allow for easy, cost-effective, large volume and repeated collection with a non-invasive procedure that can be performed by individuals with limited training, including patients, outside of the hospital. Additionally, no blood cell lysis is required that may diminish antigen availability and detection [7]. 

As urine is an ultra-filtrate of blood, most plasma or serum proteins could potentially be detected in urine at low-molecular weights and are suitable to be used for investigating the pathological process of not only kidney diseases but also systemic diseases [8]. Serum proteins are filtered through glomeruli according to their sizes and charges, and the abundant proteins including albumin, immunoglobulin light chain and transferrin are reabsorbed in proximal renal tubules [9]. Normal protein excretion in healthy individuals is less than 150 mg/day and over 6000 proteins in total are estimated in normal human urine to date [10]. A study reported approximately 30% of urinary proteins originated from the plasma proteins [11], while another more recent study observed a total of 2940 (47.7%) of the gene products identified in urine overlapped with those in plasma (81.1%) [10]. This study also showed the presence of proteins from 44 tissues in the urine, with the brain, stomach and colon being the tissues with maximum numbers of highly expressed proteins detected in urine, both at protein and mRNA levels [10]. For infections of organs other than the urinary tract, antigens from pathogens or antibodies against the pathogens found in blood could also be detected in urine, for instance *Helicobacter pylori* [12], dengue virus [13] and *Streptococcus pneumonia* [14], suggesting that the antigens and antibodies are also filtered from blood into the urine. In addition, some of the cell-free nucleic acids in plasma and blood resulting from the breakdown of DNA released from dying human cells and microorganisms pass through the kidney and are excreted in urine as transrenal DNA [15]. These DNA fragments have become popular targets for polymerase chain reaction (PCR)-based detection of pathogens, such as *Mycobacterium tuberculosis* [15] and cancers, such as breast cancer [16]. Therefore, the presence of plasma or blood proteins and transrenal DNA confers a large potential diagnostic value upon urine for disease or infection detection and monitoring. 

Collection of saliva is even more convenient than that of urine since it can be collected anytime and anywhere which does not even require a toilet or a place to urinate. Interstitial fluid from blood capillaries enters the salivary gland ducts where it is converted from isotonic to hypotonic fluid, forming the foundation of saliva [17]. The typical protein concentration in saliva is 0.7–2.4 mg/mL, despite the fact that there has been much variation in protein content based on collection time, sex, age and pathological situations [18]. An approximate total of 2290 proteins were reported by various studies determining the protein profiles of whole saliva [1]. Of the 2698 proteins identified in plasma, 27% (702) overlapped with salivary proteins. Rather than 60–80% total weight being dominated by immunoglobulins and albumins as occurred in plasma, the top 20 most abundant proteins were found to constitute only 40% of the salivary protein content [1]. This may promote detection of biomarkers from the remaining 60% of proteins [19]. The functions of salivary secretions include lubrication, antimicrobial processes, protection of mucosal integrity and digestion of food [1]. A study found that 7% of the total salivary proteins identified were immunoglobulins, and 58% of these were found in plasma, suggesting a leakage of these overlapped immunoglobins from plasma to saliva [1]. A significant linearity of immunoglobulin distributions between the plasma and saliva compartments (isotypes, subtypes) indicates that antibodies may be detectable in saliva with concentration reflective and linear to the plasma concentration. This explains the rationale for the development of the saliva SARS-CoV-2 [20], HIV [21] and HBV [22] antibody detection tests. On the other hand, saliva contains markers such as antigens, DNA and 16S RNA sequence that are frequently used as targets for saliva-based diagnostic kits, even though pathogens are not detectable in saliva [23]. Despite the low overlap in protein profiles, gene ontology distributions of salivary and plasma proteins are extremely similar [1]. This further confirms the relevance of salivary fluids for clinical use in disease detection and health screening.

The possible presence of DNA fragments and proteins of pathogens in urine and saliva seems encouraging for *Plasmodium* detection in these sample types. This review describes and summarizes the performance of nucleic acid- and antigen-based diagnostic methods evaluated for *Plasmodium* detection in urine and saliva samples by recruiting related articles published until 2022, mainly from PubMed. This review may also show the progress, evolvement and improvement made by researchers in the development of *Plasmodium* diagnostic methods in urine and saliva across the last 20 years.

## 2. Nucleic Acid-Based Diagnostic Methods

Nucleic acid-based malaria diagnostic methods are actively developed and used by researchers due to their high accuracy, sensitivity and specificity in detecting *Plasmodium* in patient samples. The emergence of highly sensitive technology such as quantitative PCR and digital PCR allows for the detection of *Plasmodium* at very low parasitemia or target gene copy numbers. The flexibility of designing primers to amplify conversed regions and specific target sequences permits high specificity in detecting *Plasmodium* from other microorganisms or parasites, as well as discerning the different species of *Plasmodium* present in the specimens. For saliva and urine samples, most nucleic acid-methods that had been developed, tested and reported so far were by using nested PCR and loop-mediated isothermal amplification (LAMP), whereby most of them amplified the common *Plasmodium* genes such as 18S ribosomal RNA (18S rRNA). The nucleic acid-based approaches used for *Plasmodium* detection in urine and saliva samples across the recent 20 years are summarized in Table 1.

### 2.1. Nested PCR

Nested PCR (nPCR) has been shown to be more sensitive and superior to microscopy in detecting *Plasmodium*, especially co-infections of *Plasmodium* species, in circulation. The detection of *Plasmodium falciparum* (*Pf*) in saliva and urine samples using nested PCR was first reported by Mharakurwa and team [24] in 2006, whereby they managed to amplify the merozoite surface protein 2 (MSP2) and dihydrofolate reductase (DHFR) region of *Pf* from saliva and urine samples with a geometric mean parasitemia of 775 asexual parasites/μL. The sensitivity and specificity of the method were not mentioned by the authors though. Another study on Gambians which also used nPCR but amplified the 18S rRNA region of *Pf* achieved sensitivity and specificity of 73% and 97%, respectively, for saliva samples, and sensitivity and specificity of 32% and 98%, respectively, for urine samples when compared with results obtained from microscopic examination [25]. The sensitivity of *Pf* detection increased to 82% for saliva samples with parasite density ≥1000 parasites/μL, which was claimed to be the level of parasitemia seen in most patients with malaria in the Gambia and other malaria-endemic areas. 

The use of this approach was extended to detection of other *Plasmodium* species such as *Plasmodium vivax* (*Pv*) detection by Buppan and colleagues [26]. In comparison with microscopy results, their 18S rRNA nPCR of saliva samples possessed a sensitivity of 74.1% and 84% for *Pf* and *Pv* detection, respectively, whereas 44.4% and 34% were the detection sensitivity of *Pf* and *Pv*, respectively, in urine samples. The specificity of both nPCR of saliva and urine samples was 100% for *Pf* and *Pv* when compared with nPCR from blood samples. The geometric means of parasite density in that study were 2761 parasites/mL for *Pf* and 1248 parasites/mL for *Pv*. 

Subsequently, 18S rRNA nPCR assays from two studies which detected *Pv* and *Pf* in saliva and urine managed to achieve an increase in sensitivity of >90% relative to microscopy results [35,38]. The increase was reproduced in a more recent study employing the same method for *Pf* detection in saliva samples, by which sensitivity and specificity were 95% and 93%, respectively, when referring to microscopy results, while corresponding values were 82% and 99% when blood nPCR was taken as reference standard [30]. Similar to the study on Gambians, this study showed an 85% sensitivity relative to both microscopy and blood nPCR when parasitemia were 1000–10,000 parasites/μL and achieved 100% sensitivity with ≥10,000 parasites/μL.

Nested PCR was then improved in order to enhance the sensitivity and specificity of *Plasmodium* detection. Instead of amplifying 18S rRNA which is present at four to eight copies in each parasite nucleus, mitochondrial cytochrome b gene located in small mitochondrial genomes (mtDNA) which span approximately 6 kb with a copy number ranging from 30 to 100 per parasite was targeted [27]. In addition to the higher copy numbers of mitochondrial cytochrome b gene that could increase the diagnostic sensitivity of *Plasmodium*, the lower divergence level of this gene sequence may also enable discrimination of *Plasmodium* species. In a study targeting mitochondrial cytochrome b gene, the overall detection limit of the nPCR assay was 10 copies/μL (150 copies) for all five *Plasmodium* species [27]. In comparison with blood nPCR, *Pf* and *Pv* detection in saliva samples had sensitivities of 74.2% and 79.2%, respectively, which were superior to those of 18S rRNA nPCR (*Pf*: 52.8%; *Pv*: 61.0%). Likewise, higher sensitivity was also seen in mitochondrial cytochrome b gene nPCR of urine samples, whereby the sensitivity was 55.1% for *Pf* and 53.3% for *Pv* detections as compared with those of 18S rRNA (*Pf*: 25.8%; *Pv*: 14.3%). The specificity of this assay in detection of *Pf* and *Pv* from saliva and urine samples ranged from 97.5 to 100%, similar to that of 18S rRNA nPCR. For *Pv*, the positive detection rates by mitochondrial cytochrome b gene nPCR of saliva reached 100% even when the parasite density was <1000 parasites/μL, whereas the assay only gave a maximum positive rate of 75% for this level of parasite density in urine, and remained almost unchanged despite increasing parasitemia. For *Pf*, positive detection rates in saliva achieved >80% even at parasitemia <1000 parasites/μL while positive rates in urine were significantly correlated with parasite density albeit the maximum positive rates achieved was 75%. This PCR assay had also shown its ability to detect *Pf* and *Pv* in saliva and urine samples with submicroscopic parasitemia. This PCR assay were reproduced on patients in southeastern Iran with higher performance, with saliva and urine samples each having a specificity of 97% and sensitivity of 91% and 70%, respectively [29]. However, the study used microscopy results as reference standard. Additionally, no correlation between parasite density and positive results of saliva and urine nPCR was observed. 

Another mitochondrial gene, mitochondrial cytochrome c oxidase III (*cox3*) gene, with 20 to 150 copies per *Pf* genome was amplified for *Pf* detection in saliva and yielded 77% sensitivity when 18S rRNA nPCR of blood samples served as reference standard (Table 1) [33]. This sensitivity outraced the 62% sensitivity of 18S rRNA nPCR of saliva samples. A similar sensitivity of 68% was obtained by the same study when *var* gene acidic terminal sequence (*varATS*) was amplified in saliva even though the gene exists at ~59 copies per *Pf* genome. However, *varATS* amplification was conducted with standard PCR instead. The *cox3* assay also showed greater efficiency in detecting submicroscopic infections in saliva compared to the 18S rRNA and *varATS* assays.

An attempt using genes present at higher copy numbers in *Pf* and *Pv* was pursued with the detection of targeted species-specific consensus repetitive sequences (CRS) *Pvr47* and *Pf346*, given that *Pvr47* are present at 14 copies in *Pv* whereas *Pfr364* exists at 41 copies in *Pf* [28,39]. However, the advantage of having higher copy numbers did not seem to make the diagnostic performance of the PCR assay better than 18S rRNA nPCR because the detection rate of *Plasmodium* in saliva samples using 18S rRNA nPCR was still the highest at 87.36% (*Pv*: 86.36%; *Pf*: 100%), followed by singleplex CRS at 81% (*Pv*: 79.5%; *Pf*: 100%) and multiplex CRS PCR assay at 70.5% (*Pv*: 70.45%; *Pf*: 71.43%) [28]. Specificity for *Pv* and *Pf* detection for all three assays was 98.48% and 100%, respectively. Furthermore, correlation between detection rate of *Pv* in saliva with parasite density was significant for CRS target-based assays. Nevertheless, it is noteworthy that the CRS PCR was a one-step PCR instead of nested PCR like the one targeting 18S rRNA. 

Interestingly, one study used antimalarial resistance genes *Pf* Kelch 13 (*PfK13*) *propeller*, *Pf* dihydrofolate reductase (*Pfdhfr*) and *Pf* chloroquine resistance transporter (*Pfcrt*) as target genes for nPCR to detect *Plasmodial* DNA in saliva and urine samples [31]. The study recorded positive detection rates of 46%, 64% and 5% for *PfK13 propeller*, *Pfdhfr* and *Pfcrt*, respectively, in saliva samples when compared to those of blood samples, while only *PfK13 propeller* and *Pfdhfr* could be detected in urine samples at sensitivity of 45% and 38%, respectively. Specificity of the assays was overall ≤50%. The copy numbers and reason for utilizing these antimalarial genes as targets were not mentioned by the authors, however, these genes could be beneficial in identification of patients infected with *Plasmodium* species harboring antimalarial resistance genes and in aiding more precise treatment of malaria. This study also suggested saliva as the best alternative sample to blood for molecular diagnosis of malaria. However, an earlier study managed to achieve a higher sensitivity of 91% with similar specificity of 50% for *Pfcrt* gene detection from *Pf* in saliva samples [32].

### 2.2. Loop-Mediated Isothermal Amplification

Loop-mediated isothermal amplification (LAMP) is an isothermal nucleic acid amplification method that amplifies target genes under isothermal conditions at 65 °C and allows for visualization of real-time reaction progress with naked eyes via color change of the reactions [40]. In addition to not requiring an expensive device such as a thermal cycler, LAMP is a simple, economic and rapid method that can be completed in 30 min with a sensitivity 10–100-fold greater than conventional PCR and 500–1000 times more sensitive than antigen detection [35,36]. Considering all the advantages that can be provided by LAMP, it has become a popular method for point-of-care diagnosis of various infectious diseases including malaria especially in endemic areas with poor conditions and limited technical resources. 

The first LAMP assay for *Pv* detection in saliva samples was attempted by Singh et al. [38]. In that study, 18S rRNA of *Pv* was targeted and sensitivity and specificity of the assay were 76.3% and 94.1%, respectively, in comparison with the results of microscopy. Nonetheless, another study aiming to detect *Pf* and *Pv* in saliva and urine samples using 18S rRNA LAMP assay demonstrated a tremendous drop of overall sensitivity to 48.5% for saliva and 30% for urine when comparing to microscopy results, but the overall specificity of the two *Plasmodium* species detection was 100% for both saliva and urine samples [35]. The detection rates of 18S rRNA LAMP were considerably associated with parasite density in blood, with maximum detection rates of 64.7% for saliva and 52.9% for urine. After that, Modak and team [36] tried to upgrade the LAMP assay by targeting mitochondrial cytochrome oxidase subunit 1 gene and using saliva samples without nucleic acid purification. Saliva eluted from the sample collectors was directly subjected to LAMP and *Pf* was successfully detected. However, actual patient saliva samples were not tested as the diagnostic performance was only evaluated on normal saliva spiked with *Pf* at a parasite density of ~4255 parasites/μL. 

### 2.3. Other Nucleic Acid-Based Methods

In addition to nPCR and LAMP, a more recent study tried to improve the detection of *Pf* and *Pv* in saliva by using droplet digital PCR (ddPCR), with concurrent evaluation using quantitative PCR (qPCR) [37]. The ddPCR targeting *Pf346* and *Pvr47* managed to amplify 76% of *Pf* and 57% of *Pv* in saliva samples that were ddPCR-positive in blood, while overall sensitivities of ddPCR in saliva was 73% (Table 1). The sensitivities obtained from ddPCR, however, were slightly lower than those from qPCR although the overall specificity of ddPCR (100%) was higher than that of qPCR (55%). It was also found that ddPCR was slightly more excellent than qPCR in picking up mixed infections. In ddPCR, the limit of detection of the *Pvr47* and *Pfr364* assays in blood samples was 0.1–0.9 parasites/μL for *Pf* and 0.9–2.7 parasites/μL for *Pv*.

*Plasmodium* detection in saliva and urine using real-time nucleic acid sequence-based amplification (QT-NASBA) was also attempted. Real-time QT-NASBA is a method incorporating RNA extraction, amplification of the RNA target and an internal control, and end point detection of amplification products by electrochemiluminescence (ECL) [41] or molecular beacon [42]. Real-time QT-NASBA targeting 18S rRNA was first modified from qualitative NASBA for *Plasmodium* detection by Schoone et al. [41] and the assay gave a sensitivity of 10–50 parasites/mL. Schneider et al. [42] also proved the detection limit of 20 parasites/mL for 18S rRNA real-time QT-NASBA, which was comparable with that of real-time quantitative PCR (QT-PCR). However, real-time QT-NASBA was preferred over real-time QT-PCR due to the advantages of real-time QT-NASBA, including faster turnaround time, relatively easy RNA extraction and permitting the use of finger-prick blood samples [42]. Real-time QT-NASBA also showed a detection limit down to 0.02 gametocytes/μL in dried blood spots on filter papers when amplifying gametocyte-specific *Pfs25*-mRNA [43].

In the case of saliva and urine samples, 18S rRNA detection by real-time QT-NASBA in saliva was sensitive (80%) but the target could only be amplified in two-thirds of the urine (66.7%) [34]. On the other hand, the detection rates of *Psf16*-mRNA of *Pf* gametocytes were lower for both saliva and urine samples and, surprisingly, the sensitivity of urine (20%) was slightly higher than that of saliva (13.3%) in that study. As for *Pfs25-*mRNA, perhaps because of its higher detection limit of 1710 compared to 143 RNA copy numbers for *Pfs16-*mRNA detection, none of the saliva and urine samples showed positive results in the QT-NASBA assay. It was speculated that the low detection of *Psf16*- and *Psf25-*mRNA could be because mature gametocyte mRNA is not secreted at all, the sensitivity of the method is too low, or mRNA had undergone extracellular degradation [34].

### 2.4. Conclusion

Taken together all the results, the sensitivity of *Plasmodium* detection in saliva samples (70–80%) is higher than in urine samples (30–50%), thus saliva is more suitable to be used as a non-invasive sample for detection of DNA from *Plasmodium* species. This could be explained based on evidence showing a ~600-fold reduction in the amount of parasite DNA quantified in saliva samples compared to that in peripheral blood from infected patients, while urine had an even lower amount of parasite DNA which was ~2500-fold lesser than that in peripheral blood [25]. It was also found that there was a concentration-dependent release of parasite material from blood into the saliva, whereas the correlation was not seen between parasitemia and parasite DNA in urine [25].

Among the nucleic-acid methods that have been assessed thus far, nPCR gave a better sensitivity and 18S rRNA is still the best target gene of choice although mitochondrial genes which are present at higher copy numbers such as cytochrome b and *cox3* also seemed to potentially give great performance in *Pf* detection. However, the performance of these mitochondrial genes in LAMP for *Plasmodium* detection remains to be determined. Most of the nPCR methods, regardless of the target gene used, were able to detect *Plasmodial* DNA in saliva samples with parasitemia ≤1000 parasites/μL. nPCR is also excellent in detecting mixed infection of *Plasmodium* species [26] compared to microscopy. Interestingly, some studies recorded the ability of PCR in detecting *Plasmodial* DNA in saliva and urine samples with negative parasitemia and blood nPCR results [25,33].

Nevertheless, some challenges need to be addressed when using saliva and urine samples for nucleic acid-based detection of *Plasmodium*. In addition to the selection of target genes, DNA extraction methods and primer design are important in determining the efficacy and performance of the PCR assays for detection of low-level *Plasmodial* DNA in both saliva and urine samples. Extraction with commercial kits was found to produce higher amplicon yield than the Chelex approach and the amplicon yield was improved as the parasite density increased [24]. Primers that produced longer amplicons were also found to give lower amplicon yields [24]. 

The sensitivity of the PCR assays may also rely on the volume and preservation methods of the samples. Owing to the low level of parasite DNA present in saliva and urine, the volume of samples collected would most likely affect the yield of parasite DNA extracted and thus the sensitivity of PCR assays, especially for single assays [25,44]. Field samples preserved in ethanol showed superior performance in *Plasmodium* detection by nPCR to those kept on ice without preservatives, suggesting that preservatives may play a pivotal role in preventing microorganism contamination of field samples that may interfere with the *Plasmodial* DNA extraction [26], as well as allow for storage of samples at room temperature without extreme degradation of DNA in samples [30]. Nonetheless, storage duration of saliva kept at −20 °C or −80 °C was found to have no significant effect on qPCR and ddPCR assays [37].

Another issue that should not be overlooked is the lengthy procedure of the nucleic acid-based diagnosis of malaria, including DNA extraction and PCR, especially nested PCR. While LAMP may be able to overcome the long turnaround time of standard or nested PCR, methods that allow direct application of saliva or urine samples have not been developed. Although DNA extraction involving fewer steps was tested on saliva samples, it is still at a very initial stage and has not yet been tested on patient saliva samples [36]. Hence, more effort is required to improve the preparation time of DNA from saliva or urine samples. 

## 3. Antigen-Based Diagnostic Methods

Antigen-based diagnostic methods, or more commonly known as rapid diagnostic tests (RDTs), are frequently used to detect antigens of *Plasmodium* species in blood or plasma to complement microscopic examination of blood smears. This approach is rapid, easy-to-perform and affordable, and these advantages allow for point-of-care diagnosis of malaria by laboratory or clinical personnel regardless of experience levels in endemic rural settings and areas with limited laboratory facilities. This approach is considerably more sensitive and specific than microscopy but somewhat comparable with the nucleic-acid diagnostic methods such as PCR [45]. 

Despite being massively used on blood samples, RDTs are scarcely used or evaluated for detection of *Plasmodium* species in non-invasive specimens, including saliva and urine. RDTs of saliva have been effectively employed for self-diagnosis of the recent 2019 novel coronavirus, SARS-CoV-2 [46] and the human immunodeficiency virus [47] as well as to assess immunity to measles, mumps, rubella and hepatitis [48,49]. This approach normally uses an immuno-chromatographic system impregnated with monoclonal antibodies against *Plasmodium* species to identify the parasite antigen in the blood of infected individuals [50]. Considering the invaluable benefits of using non-invasive specimens for malaria diagnosis, several studies attempted to evaluate the performance of malaria RDTs on saliva and urine samples. The most popular target antigens detected by malaria RDTs are *Plasmodium falciparum* histidine-rich protein 2 (*Pf*HRP2) and *Plasmodium falciparum* lactate dehydrogenase (pLDH). Some studies also tried to look for other antigens or antibodies which are more suitable for detection of *Plasmodium* in saliva and urine samples, or develop new devices or approaches for malaria diagnosis in saliva and urine. The antigen-based approaches used for *Plasmodium* detection in urine and saliva samples across the recent 20 years are summarized in Table 2.

### 3.1. PfHRP2 and pLDH

Urine and saliva are among the body fluids that contain HRP2 [59]. Various results raised interest in using malaria RDTs to detect malaria in these kinds of body fluids. The first study to detect *Pf*HRP2 in urine samples was by Genton et al. [51], using *Para*Sight^R^-F test which is in a form of dipstick. The sensitivity and specificity of *Pf* detection were ~80% and ~25%, respectively, compared to both microscopy and blood PCR results. Seeing that blood-based malaria RDTs gave unsatisfactory results when they were used for malaria detection in urine, the subsequent urine detections utilized a urine-based malaria test kit (UMT) developed by Fyodor Biotechnologies Baltimore, USA, that applies recombinant monoclonal antibody to detect highly repetitive cognate polyhistidine-rich protein 2 (HRP2) and fragments that are in the urine of febrile patients [50,53]. Similar sensitivities of 80–90% but higher specificities of 80–95% were obtained [50,53]. The UMT could detect the parasites at as low as 120 parasites/μL and the positive detection rates might significantly correlate with parasite density [50,53]. Nevertheless, a study managed to achieve sensitivity and specificity comparable to UMT when employing blood-based test kit (BinaxNOW Malaria Test kit, Inverness Medical, Europe) to detect *Pf* in urine, which were 86.67% and 94.12%, respectively [54]. The use of two other blood-based RDTs CareStart^TM^ Malaria (Accessbio, USA) and Global Devices Malaria (USA) kits for *Pf*HRP-2 detection, on the other hand, gave lower sensitivities of 67–80% with >95% specificities [55]. Disappointingly, UMT does not seem to always promise consistent sensitivities and specificities as mentioned above because only a 55.4% sensitivity and 47.5% specificity relative to microscopy results were obtained in a more recent study [56]. 

Unlike urine, there are no commercially available malaria RDTs that are specifically developed for saliva purposes so far. All studies evaluated the feasible usage of saliva for malaria diagnosis by making use of the blood-based RDTs. When targeting *Pf*HRP2, a malaria ELISA kit detected *Pf* in saliva at only 43% sensitivity and 100% specificity as compared to microscopy [7]. Instead, another study detected another popular protein, pLDH, of *Pf* and obtained a higher sensitivity of 77.6% and specificity of 100% relative to microscopy [32].

An RDT which can detect *Pf*HRP2 and pLDH antigens was used in more recent studies for malaria diagnosis of saliva and urine samples. When compared to blood test results, the sensitivities were 35.2% and 57% for detection of *Pf*HRP-2/pLDH from blood- contaminated urine and saliva samples, respectively, but dropped to 7.6% and 13.3% for corresponding samples clear of blood [58]. The specificities were 100% for all types of samples. The same study also showed the limit of parasite detection of 63,150 parasites/μL for urine and 57,335 parasites/μL for saliva, as well as a significant correlation between detection rates and parasite densities. However, another study with a greatly lower parasitemia range of 105 to 7200 parasites/μL used the same RDT but was able to achieve higher sensitivities of ~70% for urine and saliva relative to results of microscopy and blood PCR [4]. Rather than 100%, the study only obtained specificities of 77.1% to 93.1% for *Pf* detection in urine and saliva samples. 

### 3.2. Other Antigens/Antibodies Approach

Apart from direct antigen detection, detection of antibodies against *Plasmodium* species antigens were also attempted for malaria diagnosis. Estevez et al. [52] assessed the use of ELISA measuring IgG antibodies against *Pf* antigens, merozoite surface protein-1 (MSP-1_19_) and apical membrane antigen (AMA-1), as another approach to detect *Pf* in saliva samples. They also compared the detection performance of *Pf* in saliva collected using a conventional method and two commercially available sampling devices. For both antigens, antibody levels in plasma significantly correlated with those in saliva collected using commercial sampling devices, AMA-1 (r^2^ range 0.93 to 0.89, *p* < 0.001) and MSP-1_19_ (r^2^ range 0.93 to 0.75, *p* < 0.001), while a weaker correlation was observed with those in saliva collected using a conventional method (r^2^ range 0.64 to 0.63, *p* < 0.01). Taking plasma test as reference standard, sensitivity and specificity of saliva antibody levels to AMA-1 ranged 64–77% and 91–100%, respectively, whereas corresponding values for MSP-1_19_ ranged 47–67% and 90–97%, respectively, over the different sampling methods. The commercial sampling devices did not seem to offer a better performance in *Pf* detection in saliva samples compared to the conventional sampling method in this study.

As mentioned above, there is no saliva-based malaria RDTs available in the market so far. In 2019, Tao et al. [57] reported an effort to develop a prototype saliva-based point-of-need lateral flow immunoassay (LFIA) test which detected *Plasmodium* sexual stage protein 17 (PSSP17) of *Pf* gametocytes, given that individuals with subclinical infection carry sexual-stage gametocytes that may serve as a parasite reservoir that drives local malaria transmission through mosquitoes. PSSP17 was selected from the 35 *Pf* proteins identified from carriers with subclinical parasitic infection using a tandem liquid chromatography mass spectrometry (LC-MS/MS) approach and validated using LC-multiple reaction monitoring (LC-MRM) and qPCR analyses. When comparing with results of microscopy, qPCR quantifying gametocyte-specific transcript *pfs25* in blood and blood 18S rRNA PCR, PSSP17 LFIA was able to detect gametocytes at sensitivities of 100%, 92% and 91%, respectively, with a detection limit of 0.7 gametocytes/μL (Table 2). In addition, trophozoites could also be detected by the assay at a sensitivity of 92% relative to microscopy results.

In fact, in addition to the RDTs mentioned above, there are several more studies trying to identify antigens or antibodies that are specific for saliva or urine detection of *Plasmodium* species, or develop new approaches to detect *Plasmodium* species in both bodily fluids. Way back in 1991, a study identified 12 *Pf* antigens that reacted against antisera produced by mice immunized with urine from malaria patients using sodium dodecyl-sulfate polyacrylamide gel electrophoresis (SDS-PAGE), indirect immunofluorescence antibody and immunoprecipitation [59]. Additionally, the antibodies present in urine from malaria patients were found to pick up at least 19 *Pf* antigens. These antigens and antibodies in urine may be candidate markers for the development of urine-based RDTs for malaria diagnosis.

A study employed a completely different approach which incorporates DNA amplification to detect enzymatic activity of biomarker, namely Rolling-Circle-Enhanced-Enzyme-Activity-Detection (REEAD) system, for detection of human *Plasmodium* species in blood [60]. The system depends on isothermal cleavage/ligation events of single DNA catalyzed specifically by the *Plasmodium* enzyme topoisomerase I and subsequent hybridization of circular ligated DNA to an immobilized primer which is then elongated in a Rolling-Circle-Amplification (RCA) reaction. A circular DNA template is converted to a ~10^3^ tandem repeat Rolling-Circle-Product (RCP) that can easily be visualized at the single-molecule level [61,62]. When combined with a droplet-microfluidics Lab-on-a-Chip platform, this system permitted sensitive, specific and quantitative detection of all human malaria causing *Plasmodium* species in single drops of unprocessed blood with a detection limit of less than 1 parasite/μL [60]. Seeing the high performance of REEAD system, another study attempted this system for saliva detection of *Plasmodium* species with some modifications in order to meet the requirements for point-of-care (POC) diagnosis, including replacing i) the pump-driven microfluidics device with a pump-free system, and ii) the microscopic readout with a direct visible colorimetric readout based on horseradish peroxidase (HRP) activity [63]. As a result, pTopI could be detected in saliva from all 35 malaria patients, whereby *Plasmodium* infections could be detected in saliva samples even from patients with a relatively low parasite concentration of 2 parasites/μL in the matched blood samples. The outcome of this study suggested that pTopI-based REEAD assay holds great promise for use in POC diagnosis of malaria using saliva as the test material. 

Biosensors are currently one of the emerging technologies that are actively developed for detection of various target chemical or biological analytes. In healthcare, a great deal of biosensors have been developed to detect pathogens and cancers, as well as to measure glucose and oxygen levels. Impedimetric biosensors gain traction in diagnostic purposes due to their high sensitivity, low cost and miniaturization capability [64]. For malaria diagnosis, various types of immunosensors for detection of *Pf*HRP2 were fabricated on the basis of direct or sandwich immunoassays, with detection limit as low as 10 fg/mL in spiked plasma and 18 fg/mL in spiked whole blood [65]. The first biosensor to detect *Pf*HRP2 in saliva samples was fabricated by Soraya et al. [64], whereby they developed an interdigitated electrode (IDE) sensor capable of impedimetric and label-free detection of *Pf*HRP2 bound to anti-*Pf*HRP2 monoclonal antibodies (MAbs) immobilized on sensor surface. They demonstrated a promising feasibility of *Pf*HRP2 detection in saliva with a detection limit of 25 pg/mL in 8 out of 11 tested samples. 

### 3.3. Conclusions

In conclusion, with the current commercially available malaria RDTs, inconsistent performance of *Pf* antigen detection in saliva and urine was observed. RDTs designed for *Pf* detection in blood samples might not be suitable to be applied on urine and saliva samples. UMTs seemed to provide more sensitive and specific *Pf* detection in urine, while studies developing RDTs specific for saliva *Pf* detection also proved the potential of saliva as an alternative specimen of choice for malaria diagnosis. 

The main challenge of RDTs is their limitation to detect infections at low parasitemia [4]. WHO has set the lowest level of detection to 200 parasites/µL of blood for field tests [66]. In a study describing the correlation between antigen concentration and parasite density, a panel of malaria-infected blood samples with a parasite density of 200 parasites/µL needed a minimum of 4 ng/mL of *Pf*HRP2 to produce 95% positive detection, while ≥45 ng/mL of pLDH was required for at least 90% positive results [67]. In addition, for malaria eradication, current diagnostics need to be improved to detect increasing numbers of asymptomatic parasite carriers. To do this, detection limit of *Pf*HRP2 diagnostic tests needs to be 1–2 logs lower than achievable by current RDTs. Additionally, false positive results may be reported due to the persistence of target antigen even after successful treatment as well as the presence of rheumatoid factor and schistosomiasis in a patient. The presence of gametocytes and cross-reactivity of the RDT with other antigens may also lead to the false positivity [54]. 

For *Plasmodium* detection in urine and saliva, the possible factors that bring about the low sensitivity of RDTs may include the low levels of parasitemia, low antigen production by the parasite owing to mutations or deletions of the gene, time of void or sample collection, presence of antibodies to HRP2 antigen and the attributes of the antibodies (monoclonal and/or polyclonal) that are impregnated on the RDT kits [7,50,51,54,68]. Higher detection sensitivity could be acquired from morning void urine than the later collected urine [51]. Parasite sequestration may also reduce antigenemia and the consequent ultrafiltration of these antigens into the urine [54]. Moreover, urine-excreted proteins may have undergone degradation or proteolytic cleavage and could be challenging to be detected by RDTs that are developed for the detection of the intact antigen in blood [24]. The influence of sample storage methods on performance of RDT could not be determined because no study had actually performed comparison on this, despite a study which reported a higher sensitivity in *Pf* detection using whole saliva (77.9%) compared to supernatant of spun saliva (48.4%) that had been stored at 4 °C for 24 h prior to centrifugation [35]. Although the usage of commercially available saliva collection devices was described, the saliva collected in these devices did not confer better sensitivity to *Pf* detection than the urine or saliva samples stored either on ice or at −20 °C for logistic purposes, or subjected immediately to RDT after collection due to the convenience of RDT usage.

Most of the studies could not achieve satisfactory sensitivity due to the limitations of the commercially available kit used, which is intended to detect higher levels of *Pf*HRP2 in whole blood or plasma than are present in saliva and urine. Thus, development of a kit or test that is sensitive and specific enough to detect lower levels of the antigen present in saliva and urine could be a more appropriate strategy for malaria diagnostics and in epidemiological surveys. 

Some studies claimed that UMT provided a higher level of sensitivity than previously tested blood-specific RDT kits in urine specimens, with a detection limit of 120 parasites/µL and a 50% sensitivity at ≤200 parasites/µL [50]. Nonetheless, this review found that the sensitivities and specificities of UMT obtained from these studies were still below 90%. The low sensitivity and false positive results may be attributed to the factors mentioned above. Therefore, rather than detecting HRP2 with current UMT, the discovery and detection of other *Plasmodium* antigens which are more specific and abundant in urine may be necessary to overcome the abovementioned issues and develop a more sensitive UMT. For instance, a monoclonal antibody (UCP4W7), which was found by Markokpo and team, strongly reacted with *Plasmodium*-infected human urinary and cultured parasite antigens which could be a candidate for the development of a new urine-based test [69]. While a new UMT is yet to develop, effort in development of saliva-based diagnostic tests had been initiated either to detect *Pf*HRP2 using a more sensitive method such as immunosensors [64], or detection of other *Plasmodium* antigens or enzymes such as pTopI using REEAD [63] and gametocyte PSSP17 antigen using LFIA [57]. Promising sensitivities and very low detection limits were demonstrated by these new approaches, but, since they are still in the preliminary stage, they may require more modifications and validations for detection performance before they can be fabricated into RDTs and made commercially available. 

Overall, there is still no single approach that can meet all criteria that are needed for malaria diagnosis in settings with limited resources. These criteria include non-invasive, low cost, rapid, portable, easy-to-use, no requirement of sample preparation, less involvement of expert personnel, concurrent high sensitivity and specificity, and low detection limit. In fact, the low sensitivity and specificity of these diagnostic methods could be due to the inaccuracy of microscopic examination when microscopy was taken as reference standard. The inaccuracy of microscopic examination could result from the inability to determine malaria species at low parasitemia, less experienced microscopists and artifacts resembling malaria [70]. With the evidence showing the possible presence of *Plasmodium* DNA and proteins in patient’s urine and saliva, as well as the summary showing efforts in detecting *Plasmodium* in these sample types using various approaches, this review hopefully would inspire researchers to continue their efforts in discovering new targets and approaches that can fulfil the criteria mentioned above using these two sample types.

## Figures and Tables

**Table 1:** Summary of performance of various nucleic acid diagnostic methods evaluated for *Plasmodial* DNA detection in saliva and urine samples.

Nucleic-Acid Method (Target Genes)	*Plasmodium* Species Detected	Total Number of Saliva or Urine (Number of Microscopy-Positive Samples)	Geometric Mean Parasite Density in Blood (Range); Parasites/μL	Sensitivity	Specificity	Detection Limit	Correlation between Positive Detection Rates and Parasite Density	Reference
Nested PCR	*Pf*	51 (47)	775 (37 to 123,026)	NA	NA	NA	NA	[24]
(*MSP2*, *DHFR*)								
Nested PCR	*Pf*	386 (49, 50) *	1785	*Pf* ^m^	*Pf* ^m^	NA	NA	[25]
(18S rRNA)				Saliva: 73%	Saliva: 97%			
				Urine: 32%	Urine: 98%			
Nested PCR	*Pf, Pv*	120	Overall: 13,920	*Pf* ^m^	*Pf* ^b^	NA	*Pf*	[26]
(18S rRNA)		(*Pf*: 50;	(35 to 311,395) ^§^	Saliva:	Saliva:		Saliva (*r* = 0.797;	
		*Pv*: 46;	*Pf*: 2761 (35 to 217,805) ^§^	74.1%	100%		*p* = 0.055) ^t^	
		*Pf + Pv*: 4)	*Pv*: 1248 (35 to 44,520) ^§^	Urine:	Urine:			
				44.4%	100%			
				*Pv* ^m^	*Pv* ^b^			
				Saliva: 84%	Saliva:			
				Urine: 34%	100%			
					Urine:			
					100%			
Nested PCR	*Pf, Pv, Pm*	157	*Pf*: 8948	*Pf* ^b^	*Pf* ^b^	NA	NA	[27]
(18S rRNA)		(*Pf*: 60;	(35 to 217,805)	Saliva:	Saliva:			
		*Pv*: 50;	*Pv*: 3888 (35 to 44,520)	52.8%	100%			
		*Pm*: 2;		Urine:	Urine:			
		*Pf + Pv*: 5)		25.8%	100%			
				*Pv* ^b^	*Pv* ^b^			
				Saliva:	Saliva:			
				61%^b^	100%			
				Urine:	Urine:			
				14.3% ^b^	98.8%			
								
(Mitochondrial				*Pf* ^b^	*Pf* ^b^	10	*Pf*	[27]
cytochrome b gene)				Saliva:	Saliva:	copies/μL	Urine (*r* = 0.95;	
				74.2%	100%	(for each	*p* = 0.014)	
				Urine:	Urine:	species)		
				55.1%	98.7%			
				*Pv* ^b^	*Pv* ^b^			
				Saliva:	Saliva:			
				79.2%	100%			
				Urine:	Urine:			
				53.3%	97.5%			
Nested PCR	*Pf, Pv*	223	*Pf*: 21,850 (200 to 496,000)	Overall ^m^	*Pf* ^m^	NA	No	[28]
(18S rRNA)		(*Pf*: 7;	*Pv:* 4941 (320 to 61,600)	Saliva:	Saliva:			
		*Pv*: 88)		87.36%	100%			
				*Pf* ^m^	*Pv* ^m^			
				Saliva:	Saliva:			
				100%	98.46%			
				*Pv* ^m^				
				Saliva:				
				86.36%				
								
Singleplex PCR				Overall ^m^	*Pf* ^m^			
(Species-specific				Saliva: 81%	Saliva:	NA	*Pv*	
consensus repeat				*Pf* ^m^	100%		Saliva (*r* = 0.731,	[28]
sequences)				Saliva:	*Pv* ^m^		*p* = 0.039)	
				100%	Saliva:			
				*Pv* ^m^	98.46%			
				Saliva:				
				79.55%				
								
Multiplex PCR				Overall ^m^	*Pf* ^m^	NA	*Pv*	[28]
(Species-specific				Saliva:	Saliva:		Saliva (*r* = 0.774;	
consensus repeat				70.5%	100%		*p* = 0.024)	
sequences)				*Pf* ^m^	*Pv* ^m^			
				Saliva:	Saliva:			
				71.43%	99.23%			
				*Pv* ^m^				
				Saliva:				
				70.45%				
Nested PCR	*Pf, Pv*	99		Saliva:	Saliva:	NA	No	[29]
(Mitochondrial		(*Pf*: 14;		91% ^b^	97% ^b^			
cytochrome b gene)		*Pv*:46)		Urine:	Urine:			
				70% ^b^	97% ^b^			
Nested PCR	*Pf*	222 (53)	NA	Saliva:	Saliva:	NA	No	[30]
(18S rRNA)				95% ^m^;	93% ^m^;			
				82% ^b^	99% ^b^			
Nested PCR	*Pf*	(94)	24,682 (1200 to 200,000)	Saliva:	Saliva:	NA	NA	[31]
(*PfK13 propeller*)				46% ^b^	20% ^b^			
				Urine: 45% ^b^	Urine: 50% ^b^			
								
(*Pfdhfr-ts*)				Saliva:	Saliva:	NA	NA	[31]
				64% ^b^ Urine: 38% ^b^	50% ^b^ Urine: 50% ^b^			
								
(*Pfcrt*)				Saliva:	Saliva:	NA	NA	[31]
				5% ^b^ Urine: 0% ^b^	50% ^b^ Urine: 1% ^b^			
Nested PCR	*Pf*	37(33)	59,179 (2463–551,614)	Saliva:	Saliva:	NA	NA	[32]
(*Pfcrt*)				91% ^m^	50% ^m^			
Nested PCR	*Pf*	60 (60)	NA	Saliva:	NA	1 × 10^−5^	NA	[33]
(18S rRNA)				62% ^s^		ng/μL		
								
Nested PCR				Saliva:	NA	4 × 10^−7^	NA	[33]
(Mitochondrial				77% ^s^		ng/μL		
cytochrome c								
oxidase III gene)								
								
Standard PCR				Saliva:	NA	2 × 10^−6^	NA	[33]
(*varATS*)				68% ^s^		ng/μL		
LAMP	*Pv*	126	4916 (360 to 61,600) ^§^	Saliva:	Saliva:	NA	NA	[34]
(18S rRNA)		(*Pv*: 82)		76.3% ^m^	94.1% ^m^			
Nested	*Pf, Pv*	Saliva: 103	Overall: 3970.7 (120–	Overall ^b^	Overall ^b^	3.6	No	[35]
(18S rRNA)		Urine: 99	94,117)	Saliva:	Saliva:	parasites		
			*Pf*: 5020.8 (120–85,925)	89.4%	97.3%	/μL		
			*Pv*: 3672.0 (135–94,117)	Urine:	Urine:			
				71% Overall ^m^ Saliva: 92.2% Urine: 73.3%	100% Overall ^m^ Saliva: 97.4% Urine: 100%			
								
LAMP				Overall ^b^	Overall ^b^	35.9	Considerable	[35]
(18S rRNA)				Saliva: 47%	Saliva:	parasites	association	
				Urine: 29%	100% Urine: 100%	/μL		
				Overall ^m^ Saliva: 48.5% Urine: 30%	Overall ^m^ Saliva: 100% Urine: 100%			
LAMP	*Pf*	1	*Pf-*spiked saliva: 4255	NA	NA	1.5	NA	[36]
(Mitochondrial						parasites		
cytochrome oxidase subunit 1 gene)						/μL		
Quantitative PCR	*Pf*, Pv	146 (146)	NA	Saliva	Saliva	NA	NA	[37]
(*Pf346 and Pvr47*)				Overall:	Overall:			
				77% ^q^ *Pf*: 82% ^q^ *Pv*: 71% ^q^	55% ^q^			
								
Droplet digital PCR	*Pf*		NA	Saliva	Saliva	*Pf*:	NA	[37]
(*Pf346 and Pvr47*)				Overall:	Overall:	0.1–0.9		
				77%^d^ *Pf*: 82% ^d^ *Pv*: 71% ^d^	100% ^d^	parasites /μL *Pv*: 0.9–2.7 parasites /μL		
QT-NASBA	*Pf*	15 (15)	9320	Saliva:	Saliva:	143 RNA	NA	[38]
(*Pfs16*-mRNA)				20% Urine: 13.3%	100% Urine: 100%	copy numbers		
								
(*Pfs25*-mRNA)				Saliva: 0% Urine: 0%	Saliva: 0% Urine: 0%	1710 RNA copy numbers	NA	[38]
								
(18S rRNA)				Saliva: 66.7% Urine: 80%	Saliva: 100% Urine: 100%	NA	NA	[38]

*Pf—Plasmodium falciparum*; *Pv—Plasmodium vivax; Pm—Plasmodium malariae*; ^m^ Microscopy as reference standard; ^b^ Blood PCR as reference standard; ^s^ *18S rRNA* blood PCR as reference standard; * results from two microscopists; ^§^ Parasites/mL; ^t^ Tendency; LAMP—Loop-mediated isothermal amplification; ^q^ Blood quantitative PCR as reference standard; ^d^ Blood droplet digital PCR as reference standard; QT-NASBA—Real-time nucleic acid sequence-based amplification; NA—Not available; Ref—Reference.

**Table 2:** Summary of performance of various antigen diagnostic methods evaluated for *Plasmodium* species detection in saliva and urine samples.

Antigen-Based Diagnostic Methods (Target Antigen)	*Plasmodium* Species Detected	Total Number of Saliva or Urine (Number of Microscopy-Positive Samples)	Geometric Mean Parasite Density in Blood (Range); Parasites/μL	Sensitivity	Specificity	Detection Limit	Correlation between Positive Detection Rates and Parasite Density	Reference
Para*Sight*^R^-F Test Dipstick (*Pf*HRP-2)	*Pf*	112 (*Pf*: 73)	NA	Urine: 80.8% ^m^ 81.8% ^b^	Urine: 25.6% ^m^ 26.1% ^b^	NA	NA	[51]
Malaria Antigen ELISA kit (Cellabs) (*Pf*HRP-2)	*Pf*	40 (30)	NA	Saliva: 43% ^m^	Saliva: 100% ^m^	0.001%	NA	[7]
Optimal-IT dipsticks (pLDH)	*Pf*	144 (130)	59,179 (2463–551,614)	Saliva: 77.6% ^m^	Saliva: 100% ^m^	NA	NA	[32]
ELISA (IgG to AMA-1)	*Pf*	Tanzania: 53 The Gambia: 200	NA	Tanzania Saliva (Oracol): 76.7% ^p^ Saliva (Orasure): 64% ^p^ The Gambia Saliva: 68% ^p^	Tanzania Saliva (Oracol): 100% ^p^ Saliva (Orasure): 92.9% ^p^ The Gambia Saliva: 91% ^p^	NA	Oracol-finger-prick: r^2^ = 0.89; *p* = < 0.001 Orasure-fingerprick: r^2^ = 0.93; *p* = < 0.001	[52]
(IgG to MSP-1_19_)				Tanzania Saliva (Oracol): 46.7% ^p^ Saliva (Orasure): 66.7% ^p^ The Gambia Saliva: 53% ^p^	Tanzania Saliva (Oracol): 97.4% ^p^ Saliva (Orasure): 90% ^p^ The Gambia Saliva: 94.2% ^p^	NA	Oracol-finger-prick: r^2^ = 0.75; *p* = < 0.001 Orasure-fingerprick: r^2^ = 0.94; *p* = < 0.001	[52]
UMT dipstick (*Pf*HRP-2)	*Pf*	195 (80)	62,778.9 (60 to 792,600)	Urine: 83.75% ^m^	Urine: 83.48%^m^	120 parasites /μL	NA	[50]
UMT dipstick (*Pf*HRP-2)	*Pf*	1691 (341)	NA	Urine: 79% ^m^	Urine: 89% ^m^	NA	Yes r^2^ and *p* value: NA	[53]
BinaxNOW Malaria Test kit (*Pf*HRP-2)	*Pf*	111 (60)	NA	Urine: 86.67% ^m^	Urine: 94.12% ^m^	NA	NA	[54]
CareStart^TM^ Malaria kit (Accessbio, USA) (*Pf*HRP-2)	*Pf*	125(100)	3575 (24 to 471,556)	Urine: 67.1% ^m^	Urine: 95.2% ^m^	NA	NA	[55]
								
Global Devices Malaria (USA) (*Pf*HRP-2)				Urine: 80% ^m^	Urine: 100% ^m^	NA	NA	[55]
								
Malaria kit (Accessbio, USA) and Global Devices Malaria (USA) (*Pf*HRP-2)				Urine: 71% ^m^	Urine: 96% ^m^	NA	No	[55]
UMT dipstick (*Pf*HRP-2)	*Pf*	384 (224)	(40 to 38,280)	Urine: 55.4% ^m^	Urine: 47.5% ^m^	NA	NA	[56]
LFIA (PSSP17)	*Pf*	364 (364)	NA	Saliva: 100% (gametocyte) ^m^; 92% (trophozoite) ^m^; 92% *; 91% ^#^	NA	0.7 gametocytes /μL	NA	[57]
SD Bioline RDT kit (*Pf*HRP-2 and pLDH)	*Pf*	706 (312)	NA	*With blood* *contamination* Urine: 35.2% ^p^ Saliva: 57% ^p^ *Without blood contamination* Urine: 7.6% ^p^ Saliva: 13.3% ^p^	*With blood* *contamination* Urine: 100% ^p^ Saliva: 100% ^p^ *Without blood contamination* Urine: 100% ^p^ Saliva: 100% ^p^	Urine: 63,150 parasites/μL Saliva: 57,335 parasites/μL	Urine: r = 0.91, *p* = 0.004 Saliva: r = 0.95, *p* = 0.001	[58]
SD Bioline RDT kit (*Pf*HRP-2 & pLDH)	*Pf*	301 (84)	849 (105–7200)	Saliva: 74.5% ^b^ 75% ^m^ Urine: 70.7% ^b^ 67.1% ^m^	Saliva: 93.1% ^b^ 88.9% ^m^ Urine: 81.8% ^b^ 77.1% ^m^	NA	NA	[4]

*Pf—Plasmodium falciparum*; ^m^ Microscopy as reference standard; ^b^ Blood PCR as reference standard; ^p^ Plasma/Blood test as reference standard; *Pf*HRP-2*—Plasmodium falciparum* histidine-rich protein 2; UMT—Urine Malaria Test; LFIA—Lateral flow immunoassay; PSSP17—*Plasmodium* sexual stage protein 17; * Blood *pfs25* qPCR as reference standard; ^#^ Blood 18S rRNA PCR as reference standard; RDT—Rapid diagnostic kit; NA—Not available.
